# Wolf Predation on White‐tailed Deer Before, During, and After a Historically Mild Winter in Northern Minnesota

**DOI:** 10.1002/ece3.70562

**Published:** 2024-11-17

**Authors:** Thomas D. Gable, Austin T. Homkes, Joseph K. Bump

**Affiliations:** ^1^ Department of Fisheries, Wildlife, and Conservation Biology University of Minnesota St. Paul Minnesota USA

**Keywords:** deer mortality, kill rates, predation rates, winter predation, winter severity, wolves

## Abstract

In many southern boreal ecosystems of North America, wolves are the primary predators of white‐tailed deer, and white‐tailed deer are the primary prey of wolves. Furthermore, wolf–deer systems have and will continue to become more common as white‐tailed deer range continues expanding northward in North America. Despite this, there is little information on kill rates of wolves on deer (i.e., the number of deer killed per wolf per unit of time)—a fundamental metric of wolf predation on deer—and how kill rates vary with deer density, wolf density, and environmental conditions. We estimated kill rates of wolves on deer before, during, and after a historically mild winter in the Greater Voyageurs Ecosystem, Minnesota, USA. Kill rates of wolves on deer were low (0.009–0.018 deer/wolf/day) in fall, peaked in February (0.050 deer/wolf/day), and quickly declined to 0 deer/wolf/day by April. The kill rates of wolves on deer we observed in winter were some of the lowest kill rates of wolves on deer that have been documented. Wolves in the Greater Voyageurs Ecosystem appeared unable to catch and kill a sufficient number of deer to meet their daily energetic requirements during Winter 2023–2024, and thus most wolves likely lost weight during winter, a period when wolves are typically in peak physical condition. The rates of wolf predation we observed appeared to be well below those needed to decrease deer population density in the GVE. Thus, our work, in combination with numerous other studies, indicates winter conditions are the primary driver of deer population change in northern climates.

## Graphical abstract

We estimated kill rates of wolves on deer before, during, and after a historically mild winter in the Greater Voyageurs Ecosystem, Minnesota, USA. Kill rates of wolves on deer were low in fall, peaked in February, and quickly declined to 0 deer/wolf/day by April. Wolves in the Greater Voyageurs Ecosystem appeared unable to catch and kill a sufficient number of deer to meet their daily energetic requirements during Winter 2023–2024, and thus most wolves likely lost weight during winter, a period when wolves are typically in peak physical condition.

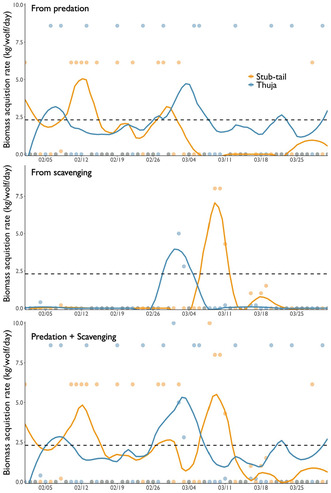

## 1 Introduction

In many southern boreal ecosystems of North America, wolves are the primary predator of white‐tailed deer, and white‐tailed deer are the primary prey of wolves (Potvin, Jolicoeur, and Huot 1988; Benson et al. 2017; Gable, Windels, and Bruggink 2017). Over the past 100–150 years, wolf–deer systems have increased as white‐tailed deer have expanded northward and outcompeted, and in some instances, replaced previously dominant ungulates such as moose and caribou (Latham et al. 2011). Much of Northern Minnesota, for instance, was a predominantly wolf–moose–caribou system that was transformed into a largely wolf–deer system due to large‐scale clear‐cut logging in the early 1900s (Cole 1987). A similar shift in wolf–prey dynamics continues to occur in Canada where the northward expansion of white‐tailed deer is being propelled by the combined forces of habitat alteration and a warming climate (Dawe and Boutin 2016; Laurent et al. 2021)—forces that are simultaneously reducing the ranges and/or populations of moose and caribou in the same areas that deer are invading (Latham et al. 2011; Weiskopf, Ledee, and Thompson 2019; Lamb et al. 2024). Thus, it is apparent that wolf–deer systems will continue to expand in North America over the coming years and decades (Kennedy‐Slaney et al. 2018; Dickie et al. 2024).

Although the relationship between wolves and deer has been studied for decades (Mech and Barber‐Meyer 2015), much of our understanding of wolf–deer dynamics is derived from cause‐specific mortality studies of deer. Such work has been undoubtedly valuable because it has allowed researchers to estimate predation rates of wolves on deer (i.e., the percent of the deer population killed by wolves annually; DelGiudice 1990; Kautz et al. 2019; Norton, Storm, and Van Deelen 2021), understand prey selection patterns of wolves (Fuller 1990; DelGiudice et al. 2006), and understand how mortality rates of deer due to wolf predation vary with environmental conditions (Nelson and Mech 1986a; Kautz et al. 2020). Despite this, there is little information on kill rates of wolves on deer (i.e., the number of deer killed per wolf per unit of time)—a fundamental metric of wolf predation on deer (Vucetich et al. 2012)—and how kill rates vary with deer density, wolf density, and environmental conditions (Gable and Gable 2019; Table 1). For instance, we are only aware of two studies (Vucetich et al. 2012; Benson et al. 2017) that have rigorously estimated kill rates of wolves on deer using ground‐based methods and by studying multiple packs (Table 1). And only one of those studies searched clusters of GPS locations from collared wolves to estimate kill rates (Benson et al. 2017), which is the best available method for estimating kill rates of cryptic large predators such as wolves (Metz et al. 2012; Elbroch, Lowrey, and Wittmer 2018; Gable and Windels 2018). The other estimates of kill rates have been based on small sample sizes (often a single pack) and aerial observations of wolves fitted with very high frequency (VHF) collars, the latter of which almost certainly underestimates kill rates of wolves on deer (Vucetich et al. 2012).

**TABLE 1:** All estimates, to our knowledge, of the kill rates of wolves on white‐tailed deer during winter based on a literature review. We also included the estimate from our study. We used the following search terms (and combinations of these terms) to identify relevant literature: “kill rates wolves,” “wolf deer,” “wolf predation,” “white‐tailed deer predation,” “wolves and deer,” “wolf predation of deer,” “winter kill rates wolves,” and “white‐tailed deer.”

Study	Location	Method	Period	Packs studied	Pack kill rate (kills/pack/day)	Individual kill rate (kills/wolf/day)	Biomass acquisition rate (kg/wolf/day)
Benson et al. (2017)^a^	Algonquin Provincial Park, Ontario	GPS clusters	December–March	22	0.12	0.015–0.040	~3.5
Fritts and Mech (1981)	Beltrami State Forest, Minnesota	VHF + aerial observation	December–March	20	0.12	0.03	2.9
Fuller (1989)	North‐Central Minnesota	VHF + aerial observation	January–February	5	0.22	0.047	2
Kolenosky (1972)	East‐Central Ontario	VHF + aerial observation	January–March	1	0.45	0.056	3.67
Mech and Frenzel (1971)^b^	Superior National Forest, Minnesota	VHF + aerial observation	Winter	1	NA	NA	0.6^b^
Mech and Karns (1977)^b^	Superior National Forest, Minnesota	VHF + aerial observation	Winter	NA	NA	NA	2.6^b^
Mech (1977)^b^	Superior National Forest, Minnesota	VHF + aerial observation	Winter	4^b^	NA	NA	1.6–3.6^b^
Stenlund (1955)^c^	Superior National Forest, Minnesota	Anecdotes	Winter	UNK	0.25^c^	NA	4.5
This study	Greater Voyageurs Ecosystem, Minnesota	GPS clusters	February–March	2	0.21	0.033	1.7
Vucetich et al. (2012)	Western Upper Peninsula, Michigan	VHF + ground tracking	December–April	5	0.68	0.141	6.7

^a^ Benson et al. (2017) examined predation of canids which included eastern wolves (*Canis lycaon*), coyotes ( *Canis latrans* ), and hybrids of the two species. The statistics reported here are for canids that had predominantly eastern wolf ancestry.

^b^ These estimates all appear to be from the same study. We could not access Mech and Frenzel (1971) or Mech (1977), the former of which was a report and the latter proceedings from a conference. However, Schmidt and Mech (1997) provide summary statistics from these studies. We would recommend viewing these estimates skeptically as we could find little to indicate these are rigorous estimates, and references to these kill rate estimates by Mech and coauthors in other papers give the impression that these are very coarse estimates (e.g., see how Mech and Karns (1977) discuss the Mech and Frenzel (1971) estimate). Furthermore, Schmidt and Mech (1997) state four packs were studied in the Mech (1977) conference proceedings, but the title of it was “Population trend and winter deer consumption in a Minnesota wolf pack” indicating only one pack was studied.

^c^ This is not a reliable estimate and we only included it here because this estimate exists in Schmidt and Mech (1997). However, Stenlund (1955) stated the following about this estimate: “Although no pack was followed to determine exactly how many deer were killed, it appears from scattered field notes on travel and on packs remaining in the vicinity of kills, that a pack of three wolves kills a deer every four days during the winter.”

Of particular interest, given current and expected changes in winter conditions, is how kill rates of wolves on deer during winter vary with winter conditions such as snow depth, snow conditions, and duration of snow cover. Most cause‐specific mortality studies of deer have identified a similar pattern: predation rates of wolves on deer are generally low during mild, shorter winters and relatively higher during severe, long‐lasting winters (DelGiudice 1990; DelGiudice et al. 2006; Kautz et al. 2020). Although such patterns suggest that winter kill rates of wolves on deer are largely driven by winter conditions, predation rates of wolves are often poorly correlated with wolves' kill rates because predation rates are a function of prey density, wolf density, and kill rates of wolves (Vucetich et al. 2012). Yet, some evidence indicates kill rates are largely a function of winter conditions. For instance, in a high snowfall area in the Upper Peninsula of Michigan, kill rates of wolves on deer almost tripled from the beginning of winter (0.32 deer/wolf/day) to the end of winter (0.95 deer/wolf/day), driven largely by increases in snow depth throughout winter (Vucetich et al. 2012).

If kill rates of wolves on deer are largely modulated by winter conditions, then it seems likely that biomass acquisition rates of wolves during winter in wolf–deer systems are also driven by winter conditions. However, whether differences in winter‐mediated biomass acquisition rates of wolves could influence wolf population dynamics in any meaningful way is poorly understood. Nelson and Mech (1986a) suggested “it is conceivable that litter size and pup survival could be affected by the vagaries of winter weather.” Implicit in this notion is that winter conditions influence the physical condition (fat reserves) of wolves via changes in biomass acquisition rates in the months prior to parturition—during mild winters, wolves would be in poorer physical condition and therefore have fewer pups (i.e., reduced litter size) and/or decreased pup survival and vice versa. Litter size can be a function, to a degree, of the body mass of breeding females indicating that the physical condition of certain individuals can influence reproductive success in wolves (Stahler et al. 2013). However, it is also plausible that wolves are able to find and kill enough vulnerable deer, even in mild winters, to build up sufficient fat reserves such that winter conditions do not meaningfully influence reproductive success or population dynamics. For instance, wolves in the Upper Peninsula, Michigan, USA, acquired an average of 7.7 kg/wolf/day during winter, far above what was needed for wolves to meet their daily energetic demands (estimated at 3–3.25 kg/wolf/day for wolves in that system; Vucetich et al. 2012). In other words, kill rates and biomass acquisition rates during winter in this system would have to decline substantially before wolves struggled to acquire sufficient food. These possibilities illustrate why detailed knowledge of kill rates of wolves on deer are necessary for a comprehensive understanding of how wolf–deer systems operate and function.

We present kill rates of deer by wolves in the Greater Voyageurs Ecosystem (GVE), Minnesota before, during, and after Winter 2023–2024, the mildest winter in recorded history in the GVE and much of northern Minnesota (Midwest Regional Climate Center 2024). The winter was especially notable for the lack of snow. Total snowfall during the winter was 110 cm with average monthly snow depths during December to March ranging from 0.8 to 14 cm from December 2023 to March 2024 (National Weather Service 2024). Snow depths never exceeded 30 cm during Winter 2023–2024, which stands in stark contrast to the preceding year when snow depths exceeded 30 cm for 117 days (long‐term average = 71 days/winter). The lack of snow was accompanied by unseasonably warm temperatures (avg. temperature during December 2023–March 2024 was 6°C warmer than average temperatures during that same period during 1990–2023 [National Weather Service 2024]). Thus, the data we present provide detailed insight into how mild winter conditions influence the ability of wolves to kill deer and acquire sufficient biomass, and provide a good baseline for future research that examines how winter conditions influence wolf predation on deer.

## 2 Methods

### 2.1 Study Area

The Greater Voyageurs Ecosystem (GVE) is a 2338 km^2^ southern boreal ecosystem that includes Voyageurs National Park (882 km^2^) and a similar large amount of federal, state, county, timber company, and privately owned land south of Voyageurs National Park. The landscape is typical of southern boreal forests with dense forests (deciduous, coniferous, and mixed) interspersed with abundant bogs, wetlands, and lakes. Winters in the GVE are typically cold (average monthly temperatures in January and February are −16°C and −14°C, respectively), snowy (average snowfall: 185 cm), and long (persistent snow cover generally from November to late March to early May) (National Weather Service 2024). The wolf population in the GVE has remained high and stable for decades with mean wolf densities of 60 wolves/1000 km^2^ over the past decade (Gable, Homkes, and Bump 2024). The primary prey of wolves in the GVE are white‐tailed deer with beavers as an important secondary prey during the ice‐free season (~April–October) (Gable, Windels, and Bruggink 2017; Gable et al. 2023). Deer densities in the GVE have largely fluctuated between ~2 and 5 deer/km^2^ over the past decade (Gable, Windels, and Olson 2017; Gable et al. 2023). However, deer densities have declined over the past 5 years and were at ~1.7 deer/km^2^ during 2022–2023 and 2023–2024. Wolves in the GVE were a threatened species per the United States Endangered Species Act during our study, although wolf hunting and trapping occurred in Ontario just north of and adjacent to the GVE.

### 2.2 Winter Clusters, Kill Rates, and Biomass Acquisition

We captured two wolves with rubber‐padded foothold traps in Summer 2023 and fit them with GPS collars as part of the larger research effort by the Voyageurs Wolf Project. We programmed the GPS collars on both wolves to take 1‐h fixes from February 1 to March 31, 2024 (hereafter referred to as the “winter study period”). All capture and handling of wolves were approved by the University of Minnesota's Institutional Animal Care and Use Committee (protocol: UMN 1905‐37051A). One wolf, Wolf B9T, was a yearling female in the Stub‐Tail Pack, and the other a subordinate wolf, Wolf O3S, a yearling in the Windsong Pack when collared in May 2023. Within a month of being collared, O3S dispersed from the pack and subsequently joined an existing pack (the Thuja Pack) in September 2023. Although O3S was initially a subordinate member in the Thuja Pack, he became the breeding male of the pack in early winter and remained in that role for the duration of the winter.

We searched all clusters of GPS locations from both wolves during our winter study period to identify kills and scavenging events. We identified clusters of GPS locations using the GPSeqClus package in R (Clapp, Holbrook, and Thompson 2021). We considered a group of locations to be a winter cluster when ≥ 2 locations were within 200 m of each other over a 2‐day period (Benson et al. 2017; Irvine, Cherry, and Patterson 2022; Cluff and Mech 2023). We then promptly visited all cluster locations in the field to determine whether a kill or scavenging event occurred. We distinguished between kill and scavenging events by assessing how fresh prey remains appeared relative to when wolves were first at the carcass, whether there was fresh blood in the snow or on the ground, whether there was evidence of a struggle or chase, and based on how the carcass was consumed (Wikenros et al. 2023). On average, we searched clusters 2.6 days after wolves left the cluster, and we visited kills 2.7 days after the kill occurred (range: 0.4–8 days). When we located a kill, we recorded sex and age when possible, assessed carcass utilization, and collected marrow from long bones if present.

When we located a scavenging event, we recorded the species wolves scavenged, estimated how fresh the carcass appeared (e.g., died within last few days, within last few weeks, and months ago), and tried to approximate the amount of food wolves likely secured during that scavenging event (Metz et al. 2012). Our intent was to assess how scavenging influenced the biomass acquisition rates of wolves during winter. Inevitably, we had to make assumptions to do this and each estimate varied based on the carcass type, age of carcass, and wolf behavior at the carcass. We have provided a detailed justification of our estimate of the amount of food wolves likely secured from each scavenged carcass we identified during winter in Appendix A.

We estimated kill rates of deer for each pack by dividing the number of kills found from each pack by the duration of the winter study period. We also estimated monthly kill rates of deer during February and March for each pack using the same approach. To account for the number of wolves in each pack, which can drive patterns in kill rates of packs (Schmidt and Mech 1997; Metz et al. 2011; Vucetich et al. 2012), we estimated individual kill rates by dividing the kill rate of each pack by the number of wolves in the pack. We determined pack size by deploying 13–16 remote cameras in known travel corridors in each pack territory during December 1–April 1 (see Gable, Homkes, and Bump 2023; Gable et al. 2024 for additional details about annual pack monitoring methods).

We then used kill rates of packs and pack size to estimate biomass acquisition rates of wolves (kg/wolf/day) during the winter study period. Estimating acquisition rates requires an estimate of the live weight of wolf‐killed prey and the amount of the prey's carcass that is digestible (Metz et al. 2011). Because estimating the live weight of the typical wolf‐killed deer is difficult, we used a plausible range of values (57–70 kg; Fuller 1989; DelGiudice, Mech, and Kunkel 1992; Vucetich et al. 2012) to capture the uncertainty in this regard, and then assumed that 76% of the live weight was digestible by wolves (Fuller 1989). Thus, each wolf‐killed deer represented 43–53 kg of digestible biomass. We then multiplied pack kill rates by the digestible biomass of the average wolf‐killed deer and divided by pack size (Metz et al. 2012; Vucetich et al. 2012), which yielded biomass acquisition rates of deer (kg of deer acquired/wolf/day during the winter study period). We then incorporated the biomass we estimated wolves acquired from scavenging during the winter study period (Appendix A). We did this by taking the total biomass acquired via scavenging for each pack and dividing it by the number of days in the study period and then by pack size, which yielded an estimate of the kilograms of scavenged biomass per wolf per day. To determine overall biomass acquisition rates of wolves in each pack during the winter study period, we simply added the biomass acquisition rate from kills and scavenging. We used this same approach to estimate biomass acquisition rates for each month during the study period as well. Notably, we assumed, based on the average body weight of wolves in the GVE (28 kg, Gable and Windels 2018), that wolves needed to acquire 2.3 kg/wolf/day to meet their minimum energetic requirements.

### 2.3 Kill Rates of Deer in Fall and Spring

We compared kill rates of deer during winter to kill rates during the preceding fall (September–October 2023) and the following spring (April–May 2024). During Fall 2023 and Spring 2024, we programmed GPS collars to take locations every 20 min. We then searched every cluster of GPS locations from collared wolves during these periods to identify kills and estimate kill rates. Notably, because wolves frequently kill small prey (e.g., beavers) during spring to fall in the GVE, we defined clusters differently during summer because wolves have considerably shorter handling times of small prey (Gable et al. 2023). We considered a cluster during Fall 2023 and Spring 2024 to be ≥ 2 consecutive locations ≥ 20 min apart and within a 200 m radius of one another.

To estimate kill rates in Fall 2023, we took the total number of deer killed per month by 6 packs with at least 1 GPS‐collared pack member and divided it by the total number of wolves in these 6 packs, which we determined using remote cameras (Gable et al. 2024), and then divided that number by the number of days we studied wolves in a given month. In Fall 2023, we searched clusters for all wolves for the entirety of September (30 days) and then almost all of October (29 days). In April, we searched clusters/estimated kill rates for the latter half of April (April 15 to 31st) and assumed these estimates were representative of the entire month. We did not search clusters from April 1 to the 15th because we did not want to incidentally visit dens just before, during, or just after wolves had given birth. In May, we searched clusters from three wolves in three different packs for the duration of the month. We estimated kill rates from two other wolves by searching clusters for 18 and 26 days in May.

When estimating kill rates for Fall 2023, we assumed that wolves were largely traveling in cohesive social groups. Thus, the kills found by studying a collared pack member were those made by the pack. By contrast, we assumed that all wolves were hunting and killing prey by themselves in Spring 2024 because GPS collar data (Demma, Barber‐Meyer, and Mech 2007; Demma and Mech 2009) and predation data (Gable et al. 2023; Gable et al. 2024) in northern Minnesota indicate wolves primarily travel and kill prey by themselves in spring and summer in northern Minnesota. Thus, we assumed that the kills made by collared wolves represented only those individuals' predation behavior. Notably, because none of the collared wolves studied killed adult deer in Spring 2024, this assumption has no impact on our estimates. We did not examine kill rates of deer fawns by wolves in Spring 2024 (avg. parturition date of fawns is ~May 26, Carstensen et al. 2009) because our focus was estimating kill rates of adult deer during this period.

## 3 Results

### 3.1 Winter Predation and Foraging

**FIGURE 1:**
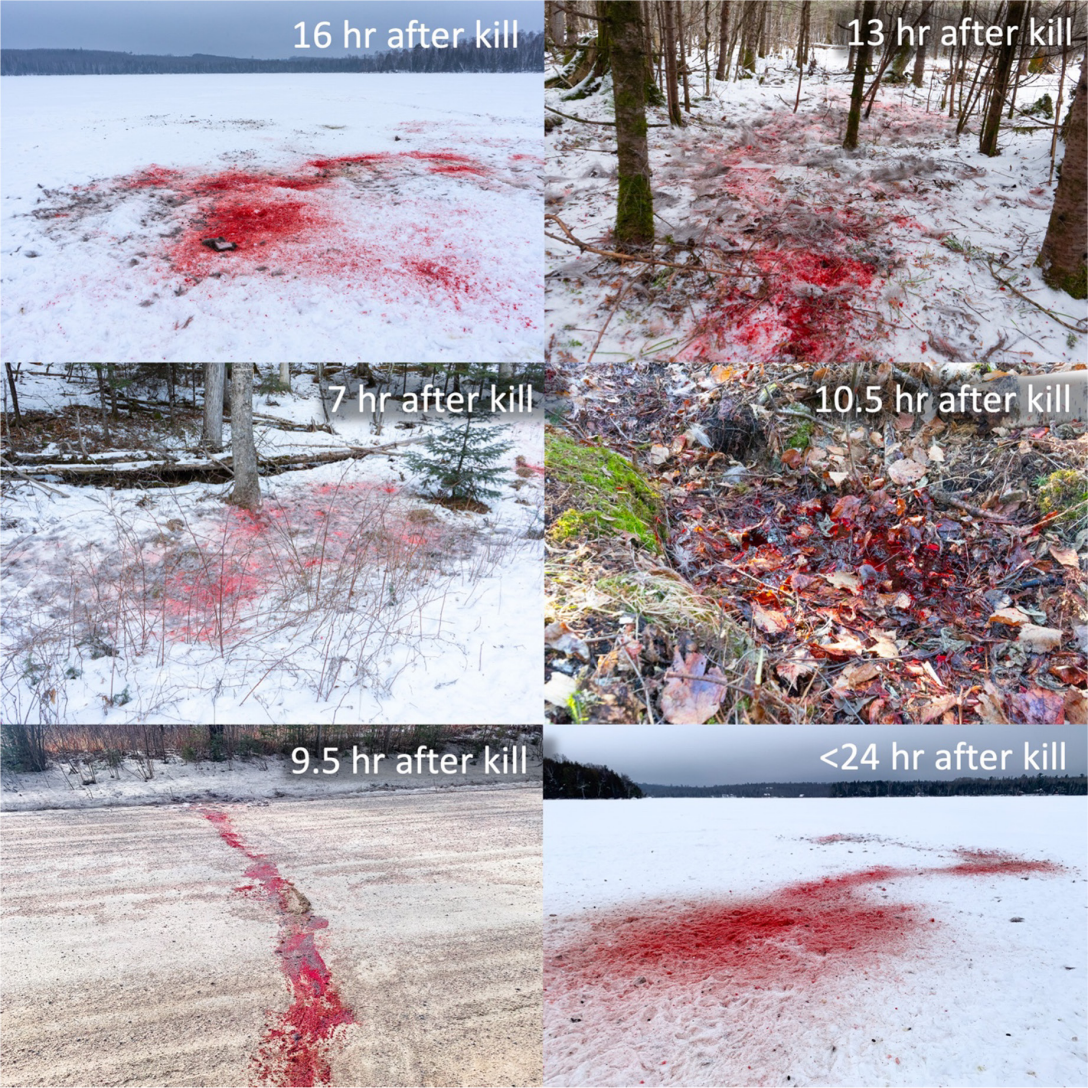
Wolves almost entirely consumed the deer they killed within short periods of time in Winter 2023–2024 leaving scant remains. Each panel here is a different kill documented in Winter 2023–2024 and the text in the upper right‐hand corner denotes how much time elapsed between when the kill occurred and when the photograph was taken. Every kill except that depicted in the bottom right was made by the Thuja Pack, which was five wolves. The bottom right was a kill made by a pack of six wolves on the lake ice in the Greater Voyageurs Ecosystem. The middle right photograph shows pooled blood of a wolf‐killed deer in a small depression. The only other remains at this kill when visited were bone fragments, hair, and rumen contents.

Based on remote camera observations, the Stub‐Tail Pack was seven wolves (a breeding pair, a yearling female, and four pups), and the Thuja Pack was five wolves (a breeding pair and three pups) during our study period (February 1–March 31). We had a collared yearling wolf in the Stub‐Tail Pack and a collared breeding male in the Thuja Pack. We searched all GPS clusters (*n* = 335) that occurred between February 1 and March 31, 2024 (60 days) from these two wolves to understand the predation behavior of their respective packs. In doing so, we identified 25 wolf‐killed deer—11 killed by the Thuja Pack and 14 by the Stub‐Tail Pack. We did not identify kills of any other species. Wolves wholly consumed the carcasses of deer they killed during this period and mean carcass utilization was 99%. All evidence we observed indicated kills were almost entirely consumed by wolves and other scavengers within a 7–24 h period (Figure 1).

Wolves scavenged carcasses of animals at 18 clusters, which equated to an estimated 194 h of scavenging. However, 96% (187/194 h) of time spent scavenging occurred in March. The Thuja Pack scavenged carcasses at eight clusters, which included five clusters where the pack scavenged the relatively scant remains of deer carcasses and then three clusters at the carcass of a moose calf that had recently died, presumably from natural causes (the wolves returned to and from the moose carcass for a 10‐day period in early March). In total, the Thuja Pack spent an estimated 80 h scavenging with a substantial proportion of that time (54%; 43 h) occurring at the moose calf carcass. The Stub‐Tail Pack scavenged prey at 10 clusters, which included one cluster at the older skeletal remains of a bull moose, one cluster at a beaver carcass exposed in the thawing ice, two at an old deer carcass dump (bones from deer that were likely killed and butchered during the November hunting season), and six clusters at the fresh carcass of an adult horse that had been dumped. In total, the Stub‐Tail Pack spent an estimated 114 h scavenging, although the majority of that time (75%; 85 h) occurred at the horse carcass.

**FIGURE 2:**
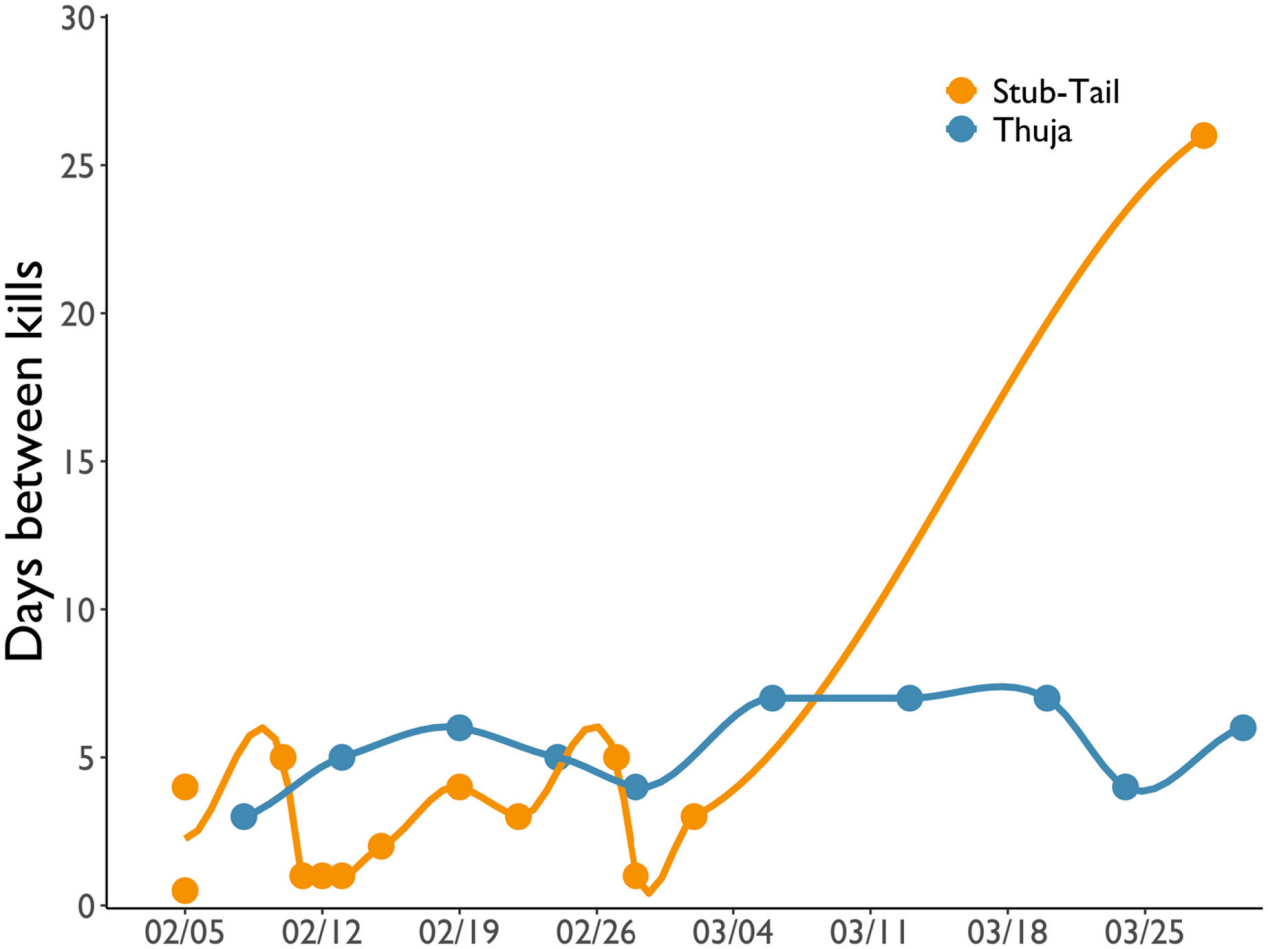
How the kill rates of wolves on deer, represented as days between kills, varied from February 1 to March 31, 2024, in the Greater Voyageurs Ecosystem, Minnesota, USA.

During February and March, the Thuja and Stub‐Tail Packs killed deer at a rate of 0.18 deer/pack/day (0.037 deer/wolf/day) and 0.23 deer/pack/day (0.033 deer/wolf/day), respectively. Kill rates of the Thuja Pack decreased slightly from 0.21 deer/day (0.041 deer/wolf/day) in February to 0.17 deer/day (0.032 deer/wolf/day) in March, whereas the kill rate of the Stub‐Tail Pack decreased dramatically from 0.41 deer/day (0.059 deer/wolf/day) in February to 0.07 deer/day (0.009 deer/wolf/day) in March—a pattern driven by the fact that the Stub‐Tail Pack went 26 days without killing a deer in March (Figure 2). These trends in kill rates were reflected in the change, or lack thereof, in the number of days between kills from February 1 to March 31, 2024.

**FIGURE 3:**
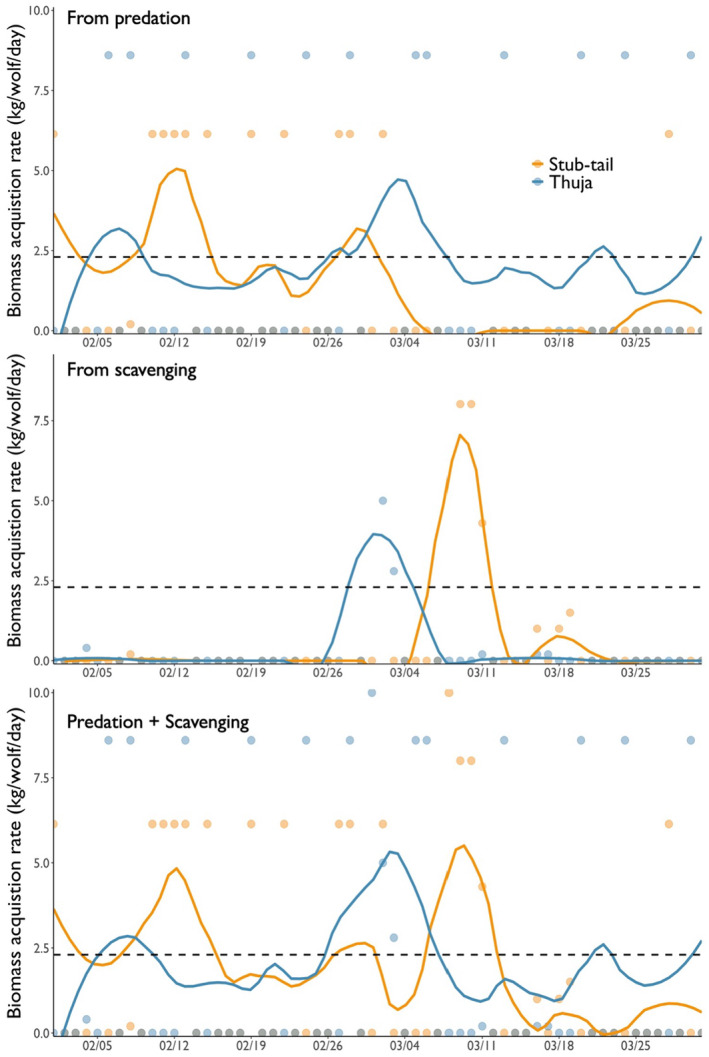
Biomass acquisition rates of wolves (kg/wolf/day) in two packs in the Greater Voyageurs Ecosystem (GVE), Minnesota, USA, from February 1 to March 31. Each point represents the biomass acquired on a given day from predation (top panel), scavenging (middle panel), and both predation and scavenging (bottom panel). We used a LOESS smoother to illustrate patterns in biomass acquisition during this period. The dashed black line represents the daily energetic requirements of wolves (2.3 kg/wolf/day) in the GVE. The spikes in biomass acquisition for both packs in early March were due to the Thuja Pack scavenging a recently deceased moose calf and Stub‐Tail scavenging a horse that had been dumped. Notably, we assumed in Panels A and C that wolves consumed all available biomass from kills (i.e., that scavengers did not remove any biomass). Although we know scavengers likely removed a meaningful proportion of biomass at kills (> 10%–20%; Vucetich et al. 2012), we used these estimates to illustrate that even if wolves consumed all biomass from kills, the average wolf was still struggling to acquire sufficient biomass to meet their daily energetic requirements for most of this period.

Wolves in the Thuja Pack acquired an estimated 1.9–2.2 kg of biomass/wolf/day and wolves in Stub‐Tail Pack an estimated 2.0–2.3 kg of biomass/wolf/day, of which 0.3 and 0.6 kg/wolf/day, respectively, were from scavenging (Figure 3). In other words, 14%–17% and 25%–29% of all biomass acquired during February and March by the Thuja and Stub‐Tail Packs, respectively, was from scavenging. Interestingly, although kill rates of deer by the Thuja Pack decreased by 14% from February to March, biomass acquisition rates of wolves in the pack increased from an estimated 1.8–2.2 kg/wolf/day in February to an estimated 2.5–2.8 kg/wolf/day in March because of scavenging (Figure 3). Contrastingly, wolves in the Stub‐Tail Pack had much higher biomass acquisition rates in February (2.5–3.1 kg/wolf/day), when kill rates were highest, than in March when biomass acquisition rates decreased by ~60% to 1.0–1.1 kg/wolf/day.

These biomass acquisition estimates assume wolves acquired all available biomass from kills they made. However, we know scavengers—mainly eagles and ravens—removed an unknown but almost certainly considerable proportion of available biomass (Vucetich et al. 2012). If scavengers consumed 10% of available biomass from kills, wolves in the Thuja and Stub‐Tail Packs would have acquired 1.7–2.0 and 1.9–2.2 kg/wolf/day during winter, respectively. If scavengers consumed 20% of available biomass from kills, wolves in the Thuja and Stub‐Tail Packs would have acquired 1.5–1.8 and 1.7–2.0 kg/wolf/day.

### 3.2 Kill Rates of Deer in Fall and Spring

**FIGURE 4:**
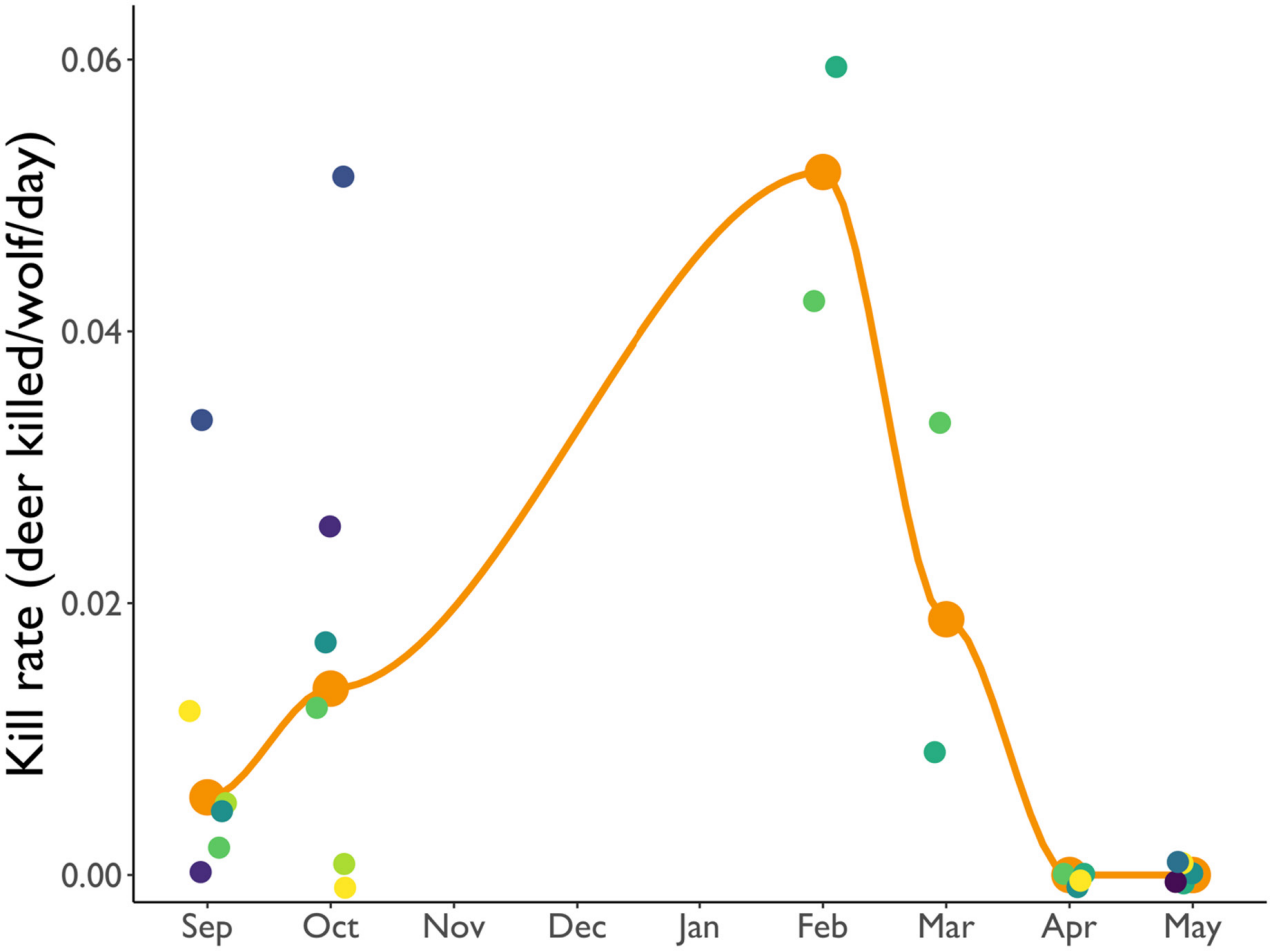
Kill rates of wolves on deer in the Greater Voyageurs Ecosystem, Minnesota from September 2023 to May 2024. The large orange circles represent average kill rates during each month. The smaller circles represent kill rate estimates for wolves in different packs from September 2023 to March 2024 and kill rate estimates for individual wolves in April and May 2024. We do not have data on kill rates from November 2023 to January 2024.

We searched 2114 clusters of GPS locations from seven wolves in six packs in September and October 2023. Cumulatively, these six packs killed six deer in September and 14 deer in October. Mean kill rates of deer by individual packs was 0.009 deer/wolf/day in September (range: 0–0.03 deer/wolf/day), and 0.018 deer/wolf/day in October (range: 0–0.05 deer/wolf/day). The total number of individuals in these six packs during this period was 35 wolves. Based on this, we estimate kill rates of deer in September and October to be 0.006 and 0.014 deer/wolf/day, respectively (Figure 4). In April and May 2024, we searched 897 clusters of GPS locations from six wolves in six packs. We did not identify any deer killed by these six wolves during this period. Therefore, we estimate kill rates of deer by wolves was 0 deer/wolf/day in both April and May (Figure 4).

## 4 Discussion

Our estimates indicate wolves in the Greater Voyageurs Ecosystem struggled and ultimately were unable to catch and kill a sufficient number of deer to meet their daily energetic requirements (2.3 kg/wolf/day; Peterson and Ciucci 2003) during the historically mild winter of 2023–2024 when snow cover was absent for most of the winter. Indeed, the kill rates of wolves on deer we observed were some of the lowest kill rates of wolves on deer during winter that have been documented (Table 1). This result is unsurprising because numerous studies have shown that the magnitude of wolf predation on deer during winter is primarily modulated by winter conditions, in particular the depth and duration of snow cover (DelGiudice 1990; Kautz et al. 2020). For instance, in a high‐snowfall area of the Upper Peninsula of Michigan where average annual snowfall is 3–4.5 m, pack kill rates of deer during February–March were almost 400% higher (0.81 deer/pack/day vs. 0.21 deer/pack/day) than those we documented (Vucetich et al. 2012). The kill rates of wolves in the GVE during the largely snow‐free winter suggest that most deer during mild winters simply are not vulnerable to wolf predation. The inability of wolves to find and kill a sufficient number of vulnerable deer to meet their daily energetic demands indicates that wolves, on average, likely lost body weight throughout winter and entered summer (pup‐rearing season)—a lean season when wolves often lose > 10%–15% of their body weight (Seal and Mech 1983; Peterson, Woolington, and Bailey 1984)—in poor physical condition. Because many aspects of wolf reproduction (e.g., litter size and pup survival) depend on the physical condition of breeding individuals (Fuller, Mech, and Cochrane 2003; Stahler et al. 2013), it seems plausible that the effects of mild winters on wolf populations could extend into the following summer (Nelson and Mech 1986a; Fuller, Mech, and Cochrane 2003).

Mid‐to‐late winter (February–April) is typically when kill rates of wolves on adult‐sized ungulates peaks because these prey are in their poorest physical condition of the year due to winter conditions and lack of high‐quality forage (Mautz 1978; Moen 1978; DelGiudice, Mech, and Kunkel 1992). Consequently, late winter is generally when wolves are in peak physical condition. Although kill rates of deer from September to May peaked in February (Figure 4), we did not document an increase in kill rates of deer by wolves from February to March. Instead, kill rates of deer remained similar throughout the winter for one pack and decreased substantially for another pack (Figure 2). We think it is likely that kill rates for most packs during mild winters remain low but static because there is little change in winter conditions (e.g., kill rates from the Thuja Pack, Figure 2), and thus vulnerability of deer throughout winter, as evidenced in trends of cause‐specific mortality of deer in wolf range during mild winters (e.g., Kautz et al. 2020). We do not entirely understand why we observed such a drastic decrease in kill rate for the Stub‐Tail Pack in March. Although the increase in scavenging could explain a small reduction in kill rate, we think it is unlikely that scavenging was the primary driver of the pack's decreased kill rate because the pack during this period was far from acquiring enough food to satiate and meet the energetic requirements of pack members. Perhaps this pack's change in kill rate is indicative of a pattern that occurs for some packs during mild winters. For instance, similar to our observations, a wolf pack in Yellowstone National Park went 20 days without killing prey in December 2023 when deep snow was absent (French 2024).

Scavenging clearly played a role in reducing the negative effects of an otherwise difficult winter for wolves. On average, wolves only acquired, assuming no biomass loss to scavengers such as eagles and ravens, 1.5–1.8 kg of biomass/wolf/day from killing deer. Yet, because scavengers likely removed at least 10%–20% of available biomass at kills (Vucetich et al. 2012), wolves likely only acquired ~1.2–1.6 kg/wolf day by killing deer—52%–70% of the 2.3 kg of biomass/wolf/day that wolves need to sustain their body weight (Peterson and Ciucci 2003). However, wolves were able to acquire an additional 0.44 kg/wolf/day from scavenging, which almost certainly minimized the average amount of weight wolves lost during winter. Interestingly, almost all the additional biomass wolves acquired from scavenging came from two carcasses: a horse carcass dumped in the Stub‐Tail territory and a moose calf that died, likely from disease, in the Thuja territory. These observations demonstrate how a single stochastic event, natural or anthropogenic, results in scavenging opportunities that can dramatically increase the biomass acquisition rates of wolves, especially when prey availability and subsequently kill rates are low.

### 4.1 Impact of Wolves on Deer Populations

Wolf predation on adult‐sized deer primarily occurs during October–April with predation generally peaking in February–April (Fuller 1990; Vucetich et al. 2012; Wehr et al. 2024). Notably, predation on adult deer from May to August is rare (Nelson and Mech 1986b; Fuller 1990; Wehr et al. 2024). During 2023–2024, we estimate that kill rates of deer in September and October 2023 in the GVE were 74% and 50% lower, respectively, than February–March 2024. Furthermore, none of the wolves we studied killed adult deer in April–May 2024, indicating that kill rates were at or very close to 0 deer/wolf/day in the spring. By using our kill rate estimates from fall, winter, and spring, and assuming kill rates in November 2023 to January 2024 were similar to average winter kill rates (0.0355 deer/wolf/day), we estimate that each wolf in the GVE killed 6.2 adult‐sized deer (i.e., deer > 3 months old) from September 2023 to May 2024. Because predation on adult deer from June to August is exceptionally rare (Nelson and Mech 1986b; Fuller 1990; Wehr et al. 2024), we can reasonably conclude that our estimate of 6.2 deer/wolf likely represents the average number of deer killed by each wolf over the course of this year. However, if kill rates from November to January were similar to those in October, we estimate each wolf killed 4.7 adult‐sized deer during the year, and if kill rates increased linearly from October to February, we estimate each wolf killed 5.5 adult‐sized deer during the year. Given all of these plausible scenarios, we estimate each wolf, on average, killed somewhere between 4.7 and 6.2 deer during the year.

Based on these estimates, we would estimate, using the most recent wolf (55.7 wolves/1000 km^2^) and deer density (prefawn densities: 1.7 deer/km^2^) estimates for the GVE (Gable, Homkes, and Bump 2024), that wolves in the GVE killed ~15%–20% of the deer population ([4.7–6.2 deer × 55.7 wolves per 1000 km^2^]/1700 deer per 1000 km^2^) during Fall 2023 to May 2024. Notably, our estimates assume lone wolves, which constitute 19%–21% of the wolf population in the GVE (Gable, Homkes, and Bump 2024), were as proficient at killing adult‐sized deer as pack wolves. Little is known about the predation behavior of lone wolves (e.g., kill rates), except for the fact that lone wolves can, at times, kill large prey (Thurber and Peterson 1993; Mech and Boitani 2003) and also go extended periods without killing prey (Stahler, Smith, and Guernsey 2006). We suspect most lone wolves are less proficient predators of deer than pack wolves, in large part because most lone wolves are young individuals (< 2.5 years old; Gese and Mech 1991) that are generally less proficient at killing prey (MacNulty et al. 2009). If so, our estimates of ~4.7–6.2 deer/wolf and ~ 15%–20% of the deer population killed likely overestimate the magnitude of wolf predation to some unknown extent.

Regardless, predation rates of this magnitude (~15%–20%) would likely be insufficient to decrease deer populations because deer populations can generally compensate for such losses. Indeed, our preliminary deer population estimates indicate that deer population density in the GVE remained fairly similar between 2022–2023 and 2023–2024. To stabilize deer populations or initiate population decline, > 25%–40% of adult females generally have to be killed (Merrill, Cooch, and Curtis 2003; McDonald Jr., Clark, and Woytek 2007; Blossey, Hare, and Waller 2024). Killing adult males generally has little effect on population change, unless done at high rates, because deer are polygynous and most deer populations where hunting occurs, like the GVE, have female‐biased sex ratios (Ueno, Kaji, and Saitoh 2010). Although all evidence we are aware of indicates wolf predation in Winter 2023–2024 would not have been sufficient to induce deer population declines, it is possible that wolf predation might influence how quickly low‐density deer populations can grow and recover after severe winters. For instance, we suspect wolf population density remains stable in the months following severe winters because most wolves are in superb physical condition from the abundance of vulnerable deer during winter. Thus, wolf populations likely do not exhibit an immediate numerical response to changes in deer populations but rather likely lag behind deer populations for a period. As a result, it is conceivable that wolf populations could reduce, even in mild winters, the speed at which deer populations recover because of a 1–2 year lag in the numerical response of wolves.

For the past century and likely much longer, there has been considerable public discussion and debate on the effects of wolves on deer populations (Olson 1938; Stenlund 1955)—a debate that intensified regionally in the last year as deer populations, and consequently hunter harvests in wolf range of Minnesota, Wisconsin, and Michigan have declined after two consecutive long, snowy winters (2021–2022 and 2022–2023; Kraker 2023; Gorniak 2024). Some believe wolves are the primary cause of deer population declines (Norton, Storm, and Van Deelen 2021) and that recreational wolf hunting and trapping are needed to ameliorate the perceived negative effects of wolves on deer populations. However, our work, in combination with numerous other studies, supports the notion that winter conditions are the primary driver of deer population change in northern climates (Kennedy‐Slaney et al. 2018; Kautz et al. 2020; Sitar and Roell 2021; Dickie et al. 2024). If wolves were the primary driver of deer populations in wolf–deer systems, then wolf predation should occur at consistently high rates even during mild winters. This would be particularly true for the Greater Voyageurs Ecosystem in Winter 2023–2024 when wolves occurred at high densities and deer at low densities. Yet, we see little evidence this is the case. Of course, there are likely certain conditions (e.g., harsh winters when wolf populations are temporarily higher than that which prey populations can support) in which wolves could, for short periods, exert top‐down control on deer populations (DelGiudice 1990; Fuller 1990). However, such periods would likely be brief (a few years) because wolf populations quickly adjust to changes in prey density (Mech and Barber‐Meyer 2015; Gable et al. 2023). Therefore, because wolves are not the primary driver of deer populations or deer hunter success, recreational hunting and trapping of wolves will likely do little to change the trajectory of deer populations or hunter success.

### 4.2 Winter 2023–2024: An Anomaly for Wolf–Deer Dynamics?

Although Winter 2023–2024 was a historically mild winter in northern Minnesota, we doubt the patterns of wolf predation we observed were entirely unique to this winter. The primary factors that increase the vulnerability of deer to wolf predation during winter are snow depth (Nelson and Mech 1986a; Olson et al. 2021) and the duration of snow cover (DelGiudice 1990; Kautz et al. 2020). Survival of deer in wolf range is generally high and mortality rates due to wolf predation generally low in years when average snow depths are low (< 30–40 cm) and snow does not persist into late March or April (DelGiudice et al. 2002; Kautz et al. 2020; Laurent et al. 2021). Furthermore, there is little to suggest that wolf predation on deer is markedly different when snow depths range from 0 to 30 cm, likely because snow depths < 30 cm do not substantially impede deer mobility (Fuller 1990; Norton, Storm, and Van Deelen 2021; Olson et al. 2021). For instance, the survival rate of female deer in north‐central Minnesota during three consecutive mild winters from 1997 to 2000 was 0.94 with a mortality rate from wolf predation around 0.03 during that period (DelGiudice et al. 2006). Over the past 18 years, the GVE has had five (28%) mild winters (2006–2007, 2011–2012, 2015–2016, 2020–2021, and 2023–2024) where average snow cover ranged from 2 to 33 cm in February and 5 to 14 cm in March, and where persistent snow cover was gone by or before March 15 (National Weather Service 2024; Midwest Regional Climate Center 2024). Given what is known about deer physiology, survival rates, and susceptibility to predation, we suspect that the patterns of wolf predation we observed are likely characteristic of wolf predation pressure during mild winters such as these.

Unfortunately, the relative dearth of information on kill rates of wolves on deer (Table 1) limits our ability to evaluate and understand how typical our results are for mild winters as well as how sensitive wolf predation patterns are to changes in winter conditions. Surprisingly, of the relatively few kill rate estimates that exist, most are based on small sample sizes (Table 1) and methods that almost certainly underestimated kill rates (e.g., VHF collars and aerial observations; Vucetich et al. 2012). In fact, most of our understanding of wolf predation on deer during winter comes from cause‐specific mortality studies of deer. While such studies are useful for understanding wolf–deer–winter dynamics and estimating the predation rate of wolves on deer, they are inherently limited in their ability to understand, measure, and quantify changes in other metrics of predation (e.g., kill rates) because they are “prey‐centric”. For a more complete and more detailed understanding of this dynamic, we think there is a need for more “wolf‐centric” research that details kill rates on deer during winter and in doing so, provides the mechanistic link between patterns in deer survival and winter conditions as well as a better understanding of wolf–deer interactions. Furthermore, as the climate continues to change, milder winters will become a more common occurrence (Laurent et al. 2021), deer will continue their northward expansion in North America (Dawe and Boutin 2016; Kennedy‐Slaney et al. 2018), and the area of wolf–deer sympatry will increase. How increasingly milder winters will influence the functional and numerical responses of wolves to deer is not well understood—and no amount of cause‐specific deer mortality studies will be informative in this regard. Ideally, future studies would simultaneously study and measure important metrics on wolf (e.g., kill rates of deer and wolf density) and deer populations (e.g., cause‐specific mortality and habitat use) during winter to better understand the causes and consequences of changing winter conditions on predator–prey ecology.

## Author Contributions

**Thomas D. Gable:** conceptualization (lead), data curation (lead), formal analysis (lead), funding acquisition (equal), investigation (equal), methodology (equal), project administration (equal), resources (equal), supervision (equal), visualization (lead), writing – original draft (lead), writing – review and editing (equal). **Austin T. Homkes:** conceptualization (supporting), data curation (supporting), investigation (equal), methodology (equal), project administration (equal), writing – review and editing (supporting). **Joseph K. Bump:** funding acquisition (equal), project administration (equal), resources (equal), supervision (equal), writing – review and editing (supporting).

## Conflicts of Interest

The authors declare no conflicts of interest.

## Supporting information


Appendix S1.

## Data Availability

We have provided all data used in this manuscript in our submission so that editors and reviews can examine it. We will archive all data associated with this manuscript to the Data Repository of the University of Minnesota upon acceptance.
